# Estrogen Receptor Alpha (ERα) Is Involved in Resveratrol-Mediated Muscle Preservation During Mechanical Unloading in Male Rats

**DOI:** 10.3390/muscles5020023

**Published:** 2026-03-25

**Authors:** David Ayi-Bonte, Samantha Dworacek, James Madden, Jacob Evans, Ingrid E. Lofgren, Kathleen J. Melanson, Christie L. Ward-Ritacco, Marie Mortreux

**Affiliations:** 1 Department of Nutrition, University of Rhode Island, 41 Lower College Rd, Kingston, RI 02881, USAingrid_lofgren@uri.edu (I.E.L.); kmelanson@uri.edu (K.J.M.); 2 Department of Kinesiology, University of Rhode Island, Kingston, RI 02881, USA; christieward@uri.edu

**Keywords:** gravity, rats, unloading, lunar, resveratrol, nutrition

## Abstract

NASA intends to return humans to the Moon, where partial gravity will put them at risk of musculoskeletal deconditioning. Resveratrol (RSV) is a promising nutritional countermeasure that may protect muscle health during disuse; however, its efficacy and mechanism in simulated lunar gravity are unknown. Forty adult male Wistar rats underwent 14 days of normal loading or partial weight-bearing at 20% of normal loading (PWB20). Unloaded animals received daily RSV supplementation with or without an ERα antagonist to test whether ERα was required to mediate RSV benefits. Muscle function was longitudinally assessed, and a Western blot was used to quantify key signaling proteins in the soleus muscle. PWB20 led to a significant reduction in grip strength (−14.2%) associated with marked changes in electrophysiological muscle properties. RSV-supplemented animals performed better throughout the study, but not when Erα was inhibited. RSV supplementation resulted in a greater ERα phosphorylation ratio compared to PWB20 alone (3.5 vs. 1.91). These results suggest that RSV can mitigate muscle deconditioning in a lunar gravity analog and that ERα signaling is required.

## 1. Introduction

Following years of research in low-Earth orbit, space agencies are now setting their sights on exploring other planets in our solar system. Among them, NASA has the ambitious plan to send the next man and the first woman to the Moon during the Artemis missions [1]. However, the significant physiological impacts associated with prolonged exposure to lunar gravity (0.16 g) represent a major challenge to overcome to ensure mission success. Lunar gravity will greatly affect the musculoskeletal system, specifically in the weight-bearing limbs, leading to musculoskeletal deconditioning characterized by loss of muscle mass, muscle weakness, and decreased bone density [2,3]. Such deconditioning can jeopardize performance during lunar activities, but crew members may also face long-lasting consequences following their return to Earth, including reduced bone density and muscle strength, increasing the risk of fractures and injuries [4,5].

The partial weight-bearing (PWB) and hindlimb suspension (HLS) models are gold standard ground-based analogs used to investigate the effects of micro- and partial gravity on the musculoskeletal system [6,7,8,9,10]. These rodent-based models have made significant contributions to our understanding of the physiological response to space stressors such as unloading and radiation [11,12,13,14]. Although there is substantial literature on the impact of microgravity and partial gravity on the musculoskeletal system [2,15], mitigating strategies to help preserve muscle function are needed to improve astronaut performance and ensure mission success. Current strategies involve a daily exercise regimen [16]; however, they do not fully prevent deconditioning and atrophy [17]. Therefore, it is imperative to research suitable adjuncts for future missions. Nutraceuticals are attractive adjuncts that are naturally present in foods [18], easy to obtain, and can be purified. Unlike other countermeasures, they also benefit from not requiring large or specific equipment [19].

Resveratrol (RSV), a naturally occurring phytoestrogen from the stilbene family, is found in grape skin, cacao, blueberries, and nuts [20,21]. It is an effective antioxidant and anti-inflammatory agent [2,22,23] that can increase insulin sensitivity, maintain protein balance and muscle mass, increase bone density, and act as an exercise mimetic during mechanical unloading in male rats [2,24,25,26,27,28,29,30,31,32]. Recent studies revealed that female rats are more resistant to unloading-induced atrophy than males [33,34], highlighting the significant influence of biological sex on musculoskeletal adaptation. Moreover, the preservation of females’ estrous cycle is correlated with the maintenance of musculoskeletal health [35], implying that sex steroid hormones, such as estrogen, may be crucial in atrophic conditions. Thus, we hypothesize that the protective effects of RSV on skeletal muscles may be mediated by estrogen receptor α (ERα). To test this hypothesis, we used adult male rats assigned to one of four experimental groups for 14 days, defined by loading condition and treatment. Animals were either freely ambulating controls (PWB100) or unloaded at 20% of normal loading (PWB20). Among unloaded animals, one group received no treatment (PWB20), one group received daily supplementation of RSV (PWB20+RSV), and one group received RSV supplementation combined with MPP, a specific ERα antagonist (PWB20+RSV+MPP).

## 2. Results

### 2.1. Effects of RSV Supplementation and the Importance of ERα in Muscle Function

**Figure 1:**
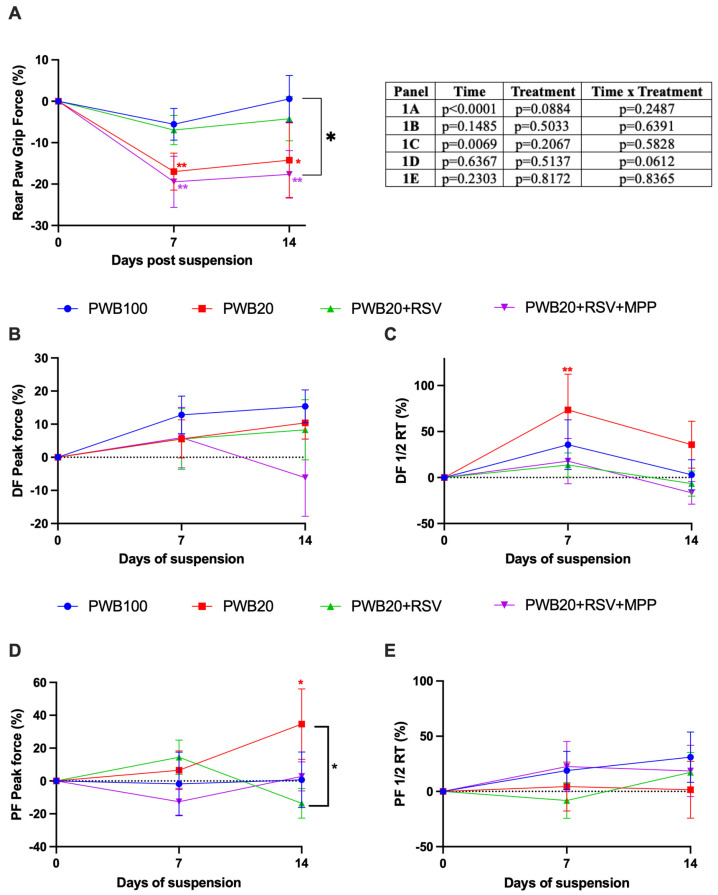
Weekly assessment of changes in rear paw grip force (**A**), nerve-stimulated force production during a dorsiflexion (DF) in panels (**B**,**C**), and a plantar flexion (PF) in panels (**D**,**E**), including changes in peak force (**B**,**D**) and a half-relaxation time (1/2 RT) (**C**,**E**) in experimental animals (n = 9–10/group). The results of the statistical analyses (2-way RM ANOVA or mixed model) are displayed in the table. Results are presented as mean ± SEM, and the result of Tukey’s post hoc tests is indicated as follows: colored *, ** represents a significant change compared to the day 0 value for the associated group (*p* < 0.05 and *p* < 0.01, respectively).

Mean rear paw grip force was significantly impacted by time (*p* < 0.0001, Figure 1A), and there were no significant differences observed between treatment groups. However, only two groups (PWB20 and PWB20+RSV+MPP) experienced a significant decline in muscle strength at 7 days (*p* = 0.009 and *p* = 0.002, respectively) compared to their baseline values, which persisted during the second week of the study. Notably, grip strength did not differ between the controls and the unloaded animals treated with RSV (PWB20+RSV) at any time during the study.

We assessed the maximal torque produced by tetanic stimulation and contractile properties in vivo using nerve-stimulated force production. The maximum peak force produced during dorsiflexion (DF) did not significantly vary throughout the experiment (Figure 1B). Contractility tests highlighted that time had a significant effect on the half-relaxation time (½ RT) during dorsiflexion (*p* = 0.007, Figure 1C), indicating ongoing transitions in myofiber types throughout the study. Indeed, ½ RT increased in all groups after 7 days, with the largest increase observed in the PWB20 group (+73.5%). Interestingly, statistical significance was not reached on day 14, despite PWB20 animals still experiencing a 35.7% increase compared to baseline.

Maximum peak force generated during plantar flexion was not impacted by time or treatment (Figure 1D). Only one group experienced significant changes in the study, with animals exposed to PWB20 displaying a significant increase between baseline and day 14 (*p* = 0.030). The ½ RT during plantar flexion did not vary throughout the study (Figure 1E).

### 2.2. Impact of Partial Gravity and Treatments on Body Weight, Food Intake, and Muscle Electrophysiological Properties

**Figure 2:**
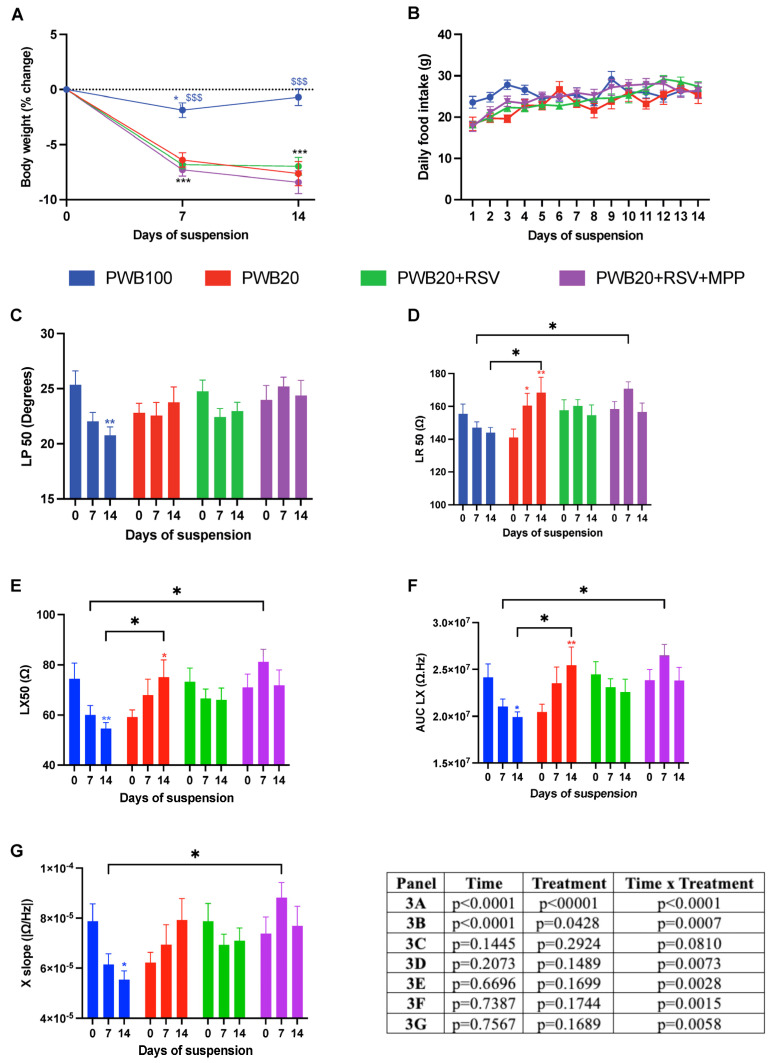
Weekly assessment of changes in body weight (**A**), daily food intake (**B**), and electrical impedance myography (EIM) parameters obtained longitudinally to the myofibers at 50 kHz, including phase (LP 50, (**C**)), resistance (LR 50, (**D**)), and reactance (LX 50, (**E**)). Weekly assessment of multifrequency EIM parameters, including the area under the curve of the reactance (AUC LX, (**F**)) and reactance-slope (X-slope, (**G**)). Results are presented as mean ± SEM, and results of Tukey’s post hoc tests following 2-way RM ANOVAs are indicated as follows: colored *, **, *** represents a significant change compared to the baseline value for the associated group (*p* < 0.05, *p* < 0.01, and *p* < 0.001, respectively), and $$$ represents a significant difference compared to all other groups at the same timepoint (*p* < 0.001).

Body weight varied significantly throughout the study (Figure 2A). Although the control group experienced a transient weight loss on day 7 (*p* = 0.048), all unloaded animals suffered a more sustained reduction in body weight. The weight loss in unloaded groups did not exceed 7.2% of their baseline, and no significant differences were observed between the treatment groups. Moreover, the major variation in body weight was observed during the first week of unloading, with no further variation during the second week of the study.

Daily food intake varied among all groups (Figure 2B, effect of time x treatment *p* = 0.0007). However, on assessment days 7 and 14, animals in all groups had a similar intake to that recorded on the first study day. The transient fluctuations in food intake were found in all groups and are unlikely to have impacted functional outcomes.

Electrical impedance myography (EIM) was used to assess muscle electrophysiological properties throughout the study. The EIM values for phase at 50 kHz (LP 50) of the PWB100 group decreased throughout the study, with a significant decline observed on day 14 (*p* = 0.005, Figure 2C). However, the values for all unloaded groups remained unchanged. Resistance at 50 kHz (LR 50) was impacted by time × treatment (*p* = 0.007, Figure 2D). Values remained stable for the PWB100 and PWB20+RSV groups, whereas animals exposed to PWB20 experienced a significant increase throughout the study (*p* = 0.0011). Animals that received RSV+MPP had significantly higher resistance compared to PWB100 on day 7 (*p* = 0.023), but not on day 14.

The longitudinal reactance (LX 50) of the control animals decreased over time, with a significant decline observed on day 14 (*p* = 0.005, Figure 2E). On the other hand, animals exposed to PWB20 showed a significant increase at 14 days (*p* = 0.031), whereas values in the PWB20+RSV+MPP group were significantly higher on day 7 compared to those in PWB100 animals (*p* = 0.020). The interaction of time and treatment had a significant impact on the area under the curve of the longitudinal reactance (AUC LX) (*p* = 0.0015, Figure 2F). Indeed, after 14 days, a significant decline was observed for PWB100 animals (*p* = 0.019), while PWB20 rats experienced a significant increase (*p* = 0.004). After 7 days, PWB20+RSV+MPP rats had significantly greater values than the PWB100 controls; however, no changes were observed over time in the PWB20+RSV and PWB20+RSV+MPP groups.

The reactance slope (X-slope) value was significantly impacted by time and treatment (*p* = 0.006). In the control animals, X slope values declined throughout the study, with a significant decrease on day 14 (*p* = 0.037, Figure 2G), whereas the values for animals in the other groups did not vary significantly. On day 7, the PWB20+RSV+MPP animals had a significantly greater X slope than control animals (*p* = 0.011).

### 2.3. Impact of RSV Supplementation and the Importance of ERα in Muscle Mass and Molecular Signaling

After 14 days of unloading, no differences were observed related to hindlimb muscle mass, except for the soleus (Table 1). Indeed, all animals exposed to PWB20 suffered comparable atrophy of the soleus muscle compared to the control animals (*p* < 0.0001), but no other hindlimb muscle was impacted.

**Figure 3:**
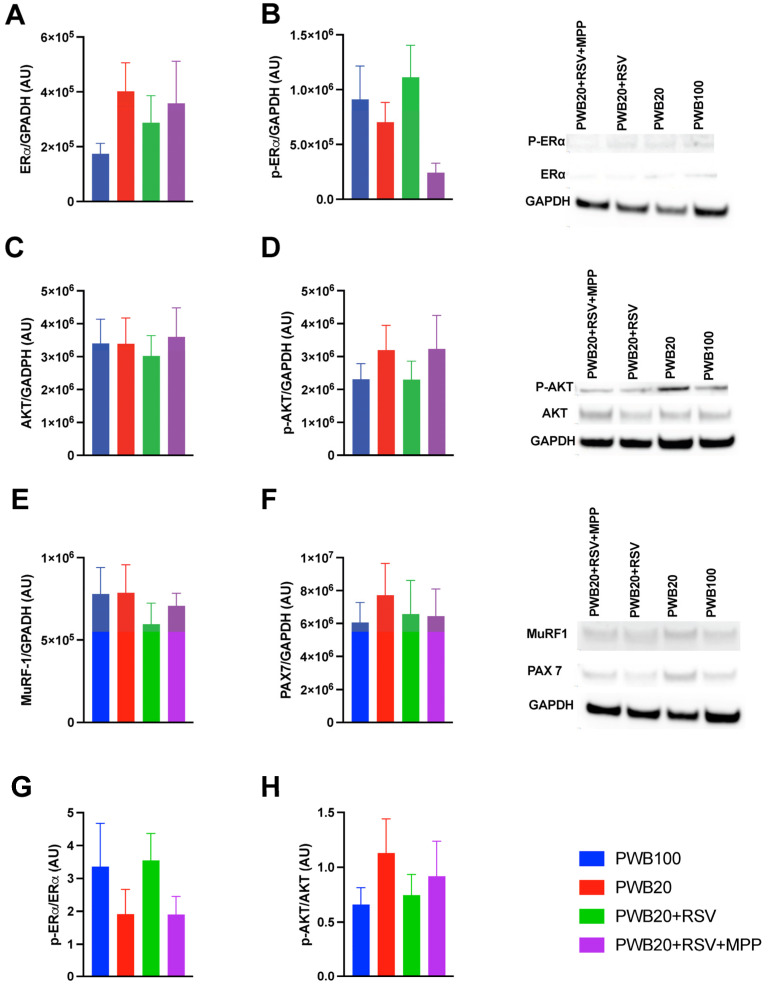
Protein semiquantification in the soleus muscle of ERα (**A**), P-ERα (**B**), AKT (**C**), p-AKT (**D**), MuRF-1 (**E**), and PAX7 (**F**) normalized to the level of GAPDH (n = 8–10 per group). Representative blot images are also displayed. Phosphorylation ratios are presented for ERα (**G**) and AKT (**H**). Results are displayed as mean ± SEM and were analyzed for normality before being tested with one-way ANOVA or Kruskal–Wallis. Abbreviations are as follows: ERα: estrogen receptor α; p-ERα: phosphorylated estrogen receptor α; AKT: protein kinase B; p-AKT: phosphorylated protein kinase B; GAPDH: glyceraldehyde-3-phosphate dehydrogenase; MuRF-1: muscle ring finger protein 1; PAX7: paired box 7; AU: arbitrary units.

We semiquantified the levels of ERα, phosphorylated ERα (p-ERα), AKT (protein kinase B), phosphorylated AKT (p-AKT), PAX7 (paired box 7), and MuRF-1 (muscle ring finger protein 1) in the soleus muscle using Western blotting (Figure 3). All groups showed similar ERα protein levels (Figure 3A, *p* = 0.746). p-Erα showed group-dependent trends that did not reach significance (*p* = 0.079), with the highest levels observed in PWB20+RSV samples and lower levels in the PWB20+RSV+MPP samples (*p* = 0.063, Figure 3B). Compared to PWB20 rats, PWB20+RSV animals had a 61.6% increase in p-ERα protein content, whereas PWB20+RSV+MPP rats displayed a 65.3% reduction. ERα activation, reflected by the phosphorylation ratio (Figure 3G), followed the same trend, i.e., lower ratios in PWB20 and PWB20+RSV+MPP groups (1.91 and 1.90, respectively) and higher ratios in PWB100 and PWB20+RSV animals (3.1 and 3.5, respectively), highlighting similar patterns despite non-significant differences.

All groups exhibited comparable AKT protein levels (*p* = 0.959, Figure 3C). In contrast to p-ERα, p-AKT levels tended to be higher in the PWB20 and PWB20+RSV+MPP groups, although this difference did not reach significance (*p* = 0.676; Figure 3D). AKT activation, reflected by the phosphorylation ratio (Figure 3H), followed a complementary pattern: lower ratios were observed in PWB100 and PWB20+RSV animals (0.66 and 0.75, respectively) compared to the PWB20 and PWB20+RSV+MPP groups (1.13 and 0.92, respectively). Finally, the levels of MuRF-1 (Figure 3E) and PAX7 (Figure 3F) did not differ significantly between groups, despite some minor variations.

## 3. Discussion

Resveratrol has been identified as a promising nutritional countermeasure against the musculoskeletal alterations induced by unloading in micro- and partial-gravity environments [2,36]. However, the specific pathway through which resveratrol provides its benefits and exerts its effect in lunar gravity has yet to be elucidated. In this study, we found that RSV supplementation of animals exposed to simulated lunar gravity prevents declines in muscle function (Figure 1). These results confirm previous reports using RSV during HLS and PWB40 (a Martian gravity analog) [2,36]. As previously reported, male rats exposed to PWB20 experienced rapid muscle deconditioning characterized by reduced grip force [8], with a 16.9% reduction observed in the first week. In our study, RSV supplementation using 200 mg/kg/day prevented this decline, but not when ERα was inhibited, suggesting that ERα plays an important role in mediating RSV signaling during unloading.

Previous studies have reported that RSV supplementation does not impact food intake or body weight at different doses (150 mg/kg/day, 400 mg/kg). Our results highlight that no changes were observed with supplementation of 200 mg/kg/day. Other teams have reported that MPP injection can have an anorexigenic effect in rats when used in doses of 50–100 µg per day [37]; however, the dose utilized in our study (25 µg/day) was selected to avoid such effects [38] and did not impact animals’ food intake, nor did it lead to loss of lean mass and body weight.

Mechanical unloading results in muscle atrophy, myofiber type switch (i.e., the creation of hybrid fibers and ultimately, fiber type transformation), and reduced neuromuscular activation [39,40], which can lead to a reduced maximal peak force in hindlimb muscles, thereby impeding muscle function [41]. Myofiber type confers different characteristics to muscles, which can be analyzed using nerve-stimulated force production tools. Indeed, fast-twitch fibers contract rapidly but are highly fatigable, resulting in shorter time to peak (TTP) and larger ½ RT compared to slow-twitch fibers [42,43]. Although we did not observe significant differences in maximal peak force throughout our study, PWB20 animals exhibited a significant increase in ½ RT during dorsiflexion after 7 days of unloading (*p* = 0.0074). This suggests a potential shift towards faster fibers, with the creation of hybrid fibers, which can result in increased fatigability. Moreover, altered myofiber composition could explain the sustained maximal force, since fast-twitch fibers have stronger force output than slow-twitch fibers [44].

In contrast to previous studies, we did not observe a significant impact of RSV supplementation on muscle wet mass (Table 1), with similar atrophy found across all animals exposed to PWB20. This conflicts with prior studies using RSV before and during hindlimb unloading (400 mg/kg/day) [2] and in simulated Martian gravity (PWB40, 150 mg/kg/day). Since musculoskeletal alterations are greater as the level of gravity decreases [45], it is possible that the dose used in our study was insufficient to mitigate disuse atrophy.

Taken together, our results suggest that 200 mg/kg/day of RSV supplementation may have had a more significant impact on contractile function than on muscle mass. Such effects have previously been observed in rats, where RSV supplementation primarily preserved muscle function despite limited impacts on muscle mass [46].

EIM is a noninvasive biomarker of muscle atrophy, as it captures the physiological alterations that occur during unloading and disuse [47,48] and can detect structural changes, including fat accumulation and edema [47]. Our results are centered around values obtained at 50 kHz, the main frequency used in muscle studies [49]. However, this frequency alone does not adequately represent the spectrum of the impedance data, and several signatures have been linked to unloading and deconditioning. Indeed, rodents exposed to microgravity, either real [50] or simulated [51], display a characteristic decline in phase values at 50 kHz. A different signature has been identified in response to simulated partial gravity [52], which may be attributed to the absence of a fluid shift in the quadrupedal unloading model, preventing the redistribution of fluids within the muscle tissue.

The partial gravity EIM signature is best captured early on during unloading (i.e., after 7 days) and characterized by stable values for phase at 50 kHz (LP50) and increased LR50, LX50, X-slope, and AUC LX values [52]. Moreover, the magnitude of the changes is directly related to the level of weight-bearing, with lower weight-bearing levels (such as PWB20) resulting in the greatest changes. We detected these changes in both the PWB20 and the PWB20+RSV+MPP groups. However, this signature was not present in the PWB100 nor in the PWB20+RSV animals (Figure 2).

The ability to detect changes during the initial phase of unloading is critical and demonstrates the important use of EIM as a biomarker for unloading-induced disuse. Importantly, this approach is also able to detect the effects of mitigating strategies [53]. The absence of the EIM signature in the PWB20+RSV group confirms that RSV supplementation prevented electrophysiological changes in the hindlimb muscles. Additionally, our results show that the addition of MPP inhibited the protective effects of RSV on muscle properties, thus suggesting a critical role of ERα in mediating RSV signaling during unloading.

We assessed changes in soleus protein levels using Western blotting (Figure 3). Since markers of muscle degradation and atrophy usually peak early during disuse [54], it was not surprising to observe comparable levels of MuRF-1 across all groups. Muscle plasticity partially relies on the activation of satellite cells (expressing Pax7) to initiate muscle repair and regeneration [55,56]. In our study, Pax7 protein content trended slightly higher in the PWB20 groups, suggesting the potential activation of repair mechanisms [55]. This is consistent with prior reports from Darr and Schultz [15] in HLS rats. Indeed, they showed that both myonuclear number and satellite cell activity were impacted by unloading duration, with increased activity occurring during the early stages of disuse. Analysis conducted in the soleus of rats after 14 days of HLS also indicated a relative increase in the number of satellite cells in the soleus muscle compared to normally loaded controls, similar to what we have reported here [34].

Cell signaling is often mediated by active phosphorylation [57]. Thus, we targeted two significant pathways involved in muscle function: ERα and AKT [57,58] and examined their phosphorylation rates. All groups showed comparable levels of non-phosphorylated proteins; however, p-ERα content was more variable, resulting in different phosphorylation rates. Indeed, compared to PWB100 controls, animals exposed to PWB20 had a 38.3% decrease in ERα phosphorylation rate, while RSV supplementation led to a 14.3% increase. Moreover, animals that received RSV+MPP had a lower ERα phosphorylation rate compared to controls. These results and trends highlight the role of RSV as a phytoestrogen [59] and help confirm the successful inhibition of the ERα signaling pathway due to the use of MPP.

AKT is a crucial signaling protein in most biological pathways, located downstream of ERα [58]. Therefore, it was surprising to observe an opposite trend. Indeed, rats in the PWB20 and PWB20+RSV+MPP groups had seemingly greater amounts of p-AKT compared to PWB20+RSV and PWB100 animals. Although surprising, it is important to note that AKT is a central signaling node regulating multiple downstream pathways, including pathways linked to muscle remodeling through the activation of mTORC1 and the ubiquitin–proteasome pathway [54]. In our longitudinal study, we were unable to assess AKT activation at earlier timepoints, and the results obtained from the soleus after 14 days of unloading may not offer an accurate picture of earlier signaling events.

This study was not without limitations. For example, we exclusively studied adult male rats, allowing us to fully block ERα signal transduction. Female rats have larger quantities of circulating estrogen, which may have prevented MPP from fully inhibiting ERα activation. Since mitigating strategies should apply to all, and considering the demonstrated importance of biological sex during disuse [33,34] and recovery from disuse [60], this work should be repeated in females. Additional considerations, such as estrous cycle monitoring and the use of an MPrP (methyl-piperidino-pyrazole) [38], another isoform of the ERα antagonist that has no agonist effect in estrogenic models, should be explored. Furthermore, we only used a single dose of RSV and a relatively short period of unloading, and our study highlights that protective effects appearing during the first week of disuse may not last throughout. Additional testing should be done on other study days to capture possible acute changes, extended past 14 days of study, and used in conjunction with different doses of RSV.

Together, our results highlight that the physiological alterations that occur in males after exposure to lunar gravity (PWB20) are comparable to those seen in rats exposed to Martian gravity (PWB40). While RSV supplementation may be a viable method for preserving muscular function, our work suggests that the ERα pathway may be one of the primary pathways through which RSV delivers its benefits.

The outcomes of this study offer insights into the pathways required to preserve muscle health and function. Leveraging these findings, coupled with additional studies in females, could lead to the improvement of mitigating strategies for spaceflight-induced muscle atrophy prevention and the development of new therapies targeting ERα. Such endeavors to preserve muscle health and function could be beneficial for astronauts and patients suffering from disuse atrophy following bed rest, injury, and metabolic diseases.

## 4. Materials and Methods

### 4.1. Animals

All experimental protocols were approved by the University of Rhode Island Institutional Animal Care and Use Committee (IACUC). Male Wistar rats (Charles River Laboratories, Wilmington, MA, USA), 14 weeks old, were obtained and housed in a temperature-controlled facility (23 ± 1 °C) with a 12-h light/dark cycle beginning at 6:00 a.m. They were housed in the facility for one week prior to the start of the experiment. Water and food were provided ad libitum, and food intake was monitored daily. Rats were housed singly in custom cages and allowed to acclimate for 48 h before being exposed to the different levels of PWB for 14 days as described by Mortreux et al. [6].

At baseline, animals were assigned to one of four groups (n = 10 per group) to ensure equal body weight distribution across the groups. The control group was normally loaded (left freely ambulating in the cage, PWB100), whereas rats in the three experimental groups were loaded at 20% of normal loading (PWB20) with a full-body harness composed of a jacket to support the front limbs and a pelvic harness to support the hindlimbs. The achieved PWB was monitored daily and calculated as described by Mortreux et al. [45]; the chain length was changed in case of a deviation greater than 5% from the target PWB level. All experiments were performed during the light phase.

Nutraceutical supplementation was provided daily at 8:00 am. Animals were given a solution prepared extemporaneously, which was composed of 0.1% stevia water (Organic Stevia, Micro Ingredients, Montclair, CA, USA), serving as the vehicle, or 200 mg/kg/day of RSV dissolved in 0.1% stevia water (Trans-Resveratrol micronized, Mega Resveratrol, Danbury, CT, USA). The solution was prepared in order for rats to receive approximately 400 μL given to them orally using a 1 mL syringe. Pharmacological treatments were administered daily using subcutaneous injections, occurring immediately before the oral supplementation. MPP (Sigma-Aldrich, St. Louis, MO, USA), a specific ERα antagonist, was dissolved in 0.1% DMSO (Sigma-Aldrich, St. Louis, MO, USA) to create a solution containing 25 μg. Animals receiving the control injection were only injected with the 0.1% DMSO vehicle.

For all procedures requiring anesthesia (maximal isometric force production, electrical impedance myography), animals were exposed to vaporized isoflurane (2.5–3.5% + oxygen) from an inhalation anesthesia system (VetEquip Inc., Livermore, CA, USA). After 14 days of PWB, animals were euthanized by CO_2_ inhalation according to IACUC guidelines.

### 4.2. Grip Force Measurements

Using a 50 N digital grip force meter (Maze Engineers, Skokie, IL, USA), both front and hindlimb grip force were measured twice weekly. The rats were positioned with their paws on a transducer bar attached to the grip force meter and gently pulled backward until they released their grip. The peak force generated was measured and recorded. This assessment was repeated three times with a short latency between trials to avoid muscle fatigue. The peak force from each trial was averaged.

### 4.3. Electrical Impedance Myography (EIM)

EIM was performed on the left gastrocnemius muscle using the mView System (Myolex, Inc., Boston, MA, USA). The rats were placed in a prone position, and the left leg was secured at a 45° angle. The fur was clipped, and intramuscular electrodes were inserted longitudinally into the triceps surae. EIM parameters were collected at 41 frequencies ranging from 1 kHz to 10 MHz. Single-frequency analysis at 50 kHz was used to assess overall muscle health, and the multifrequency analysis was focused on identifying hallmarks of unloading-induced disuse. The AUC was measured using reactance values between 5 and 500 kHz, while the reactance slope was obtained on the linear portion of the curve (100–500 kHz).

### 4.4. Maximal Isometric Force Production

Nerve-stimulated force production exerted during a plantar flexion (targeting the triceps surae) and a dorsiflexion (targeting the TA and EDL) was assessed using a dual-mode muscle lever whole rat system model 1305A (Aurora Scientific, Aurora, ON, Canada). Under anesthesia, the rats were placed in a supine position on the platform with the left paw secured to the footplate and the knee stabilized at a 90° angle. Monopolar electrodes were inserted into the popliteal fossa (for tibial nerve stimulation) and at the fibular neck (for peroneal stimulation). An initial 10 Hz twitch was first performed to ensure accurate needle positioning. This was followed by a tetanic supramaximal stimulation at 200 Hz for 200 milliseconds (ms). The force generated was measured, and force production and contractile properties were calculated using peak force, time to peak (TTP), half-relaxation time (1/2 RT), and area under the curve (AUC).

### 4.5. Tissue Collection

Following IACUC guidelines, the rats were euthanized using carbon dioxide (CO_2_), followed by cardiac puncture and decapitation at the end of the protocol. Blood obtained from the cardiac puncture was left to clot at room temperature and centrifuged to separate the serum (5 min, 4 °C, 15,000× *g*). The serum was aliquoted and stored at −20 °C for further analysis. The left hindlimb skeletal muscles (triceps surae, tibialis anterior, and extensor digitorum longus) were excised and weighed using a precision analytic scale (Mettler Toledo, Columbus, OH, USA) and fixed in 10% neutral buffered formalin (NBF, Azer Scientific, Morgantown, PA, USA) for 48 h at 4 °C before being transferred to 1× PBS and stored for further analysis. Samples obtained from the right hindlimb muscles were excised and snap-frozen in liquid nitrogen immediately and stored at −80 °C. Other organs were collected to create a biobank and allow for future analyses.

### 4.6. Muscle Immunohistochemistry

Immunohistochemical analysis was performed on the left triceps surae and incubated with antibodies targeting Laminin (ab242198, Abcam), GLUT4 (ab313775, Abcam), and counterstained with 4′,6-diamidino-2-phenylindole (DAPI, #D35471; Invitrogen). Images were observed under an epifluorescence microscope (Nikon Eclipse E600 Upright Microscope, Tokyo, Japan). To determine the myofiber cross-sectional area of the myofibers, the images were analyzed with the FIJI software (ImageJ2, v2.14.0/1.54f, NIH) using a muscle morphometry plug-in (Anthony Sinadinos using Eclipse IDE).

### 4.7. Protein Semiquantification (Western Blot)

For this assay, tissues snap-frozen in liquid nitrogen were used. Using Western blot analysis (iBlot, Invitrogen, Carlsbad, CA, USA), we determined the phosphorylation ratio of ERα and protein kinase B (AKT), as well as the levels of MuRF-1 in the muscles. MuRF-1 helps in muscle remodeling; upregulation of MuRF-1 signifies muscle atrophy. 50 mg of muscle was homogenized in a Pierce RIPA buffer (Cat: 89900, Thermo Fisher Scientific, Franklin, MA, USA) containing Halt protease inhibitor cocktail (Cat: 78429, Thermo Fisher Scientific) and centrifuged at 10,000× *g* for 10 min at 4 °C. The supernatant was collected, and protein content was determined using a BCA assay (Cat: 23227, Thermo Fisher Scientific). 80 µg of proteins was separated on 4−12% Bis-Tris plus mini protein precast gels (Thermo Fisher Scientific) and transferred to a nitrocellulose membrane using IBlot 2 Western blot transfer device (Thermo Fisher Scientific). The efficacy of the transfer was determined via Ponceau staining. Membranes were blocked in iBind solution to prevent nonspecific binding and incubated with primary antibodies targeting ERα (ab32063; Abcam), AKT (MA514916; Thermo Fisher Scientific), and MURF-1 (PA576695; Thermo Fisher Scientific). Following incubation with secondary antibody (Goat Anti-Rabbit IgG h&L, Abcam, Waltham, MA, USA), signals were developed with SuperSignal West pico plus chemiluminescent substrate (Thermo Fisher Scientific), and the immune complex was visualized using Sapphire biomolecular imager (Azure Biosystems, Dublin, CA, USA).

### 4.8. Statistical Analysis

The data or images from the Western blot were assessed with Azure Pro. All longitudinal data (e.g., body weight, achieved PWB, and food intake) were evaluated using two-way repeated-measure ANOVA or mixed-effect models with Tukey’s post hoc test. Sphericity was tested, and corrections were applied if Geisser–Greenhouse’s ε was inferior to 0.75. Cross-sectional data were analyzed using ordinary two-way ANOVA or mixed-effect models with Tukey’s post hoc test, and the results were significant at *p* < 0.05. All statistical analysis was performed using GraphPad Prism 10.0.2.

## 5. Conclusions

These results suggest that phytoestrogens such as RSV can preserve muscle function during disuse and that ERα may be critical to its action in skeletal muscles. The outcomes of this study offer insights into the role of ER and could lead to the development of targeted therapies for disuse atrophy following bed rest, injury, and metabolic diseases such as sarcopenia.

## Figures and Tables

**Table 1:** Hindlimb muscle wet mass normalized to body weight.

	PWB100	PWB20	PWB20+RSV	PWB20+RSV+MPP	*p*-Value
S	0.057 ± 0.002 ^a^	0.045 ± 0.002 ^b^	0.047 ± 0.001 ^b^	0.048 ± 0.002 ^b^	*p* < 0.0001
GP	0.662 ± 0.01 ^a^	0.637 ± 0.017 ^a^	0.628 ± 0.014 ^a^	0.632 ± 0.016 ^a^	*p* = 0.3882
TA	0.217 ± 0.004 ^a^	0.213 ± 0.006 ^a^	0.208 ± 0.003 ^a^	0.209 ± 0.005 ^a^	*p* = 0.4785
EDL	0.049 ± 0.001 ^a^	0.050 ± 0.00 ^a^	0.047 ± 0.001 ^a^	0.048 ± 0.001 ^a^	*p* = 0.1934

Multiple linear regressions using the least squares method were performed to analyze the contributions of the experimental factors (ANCOVA) to muscle wet mass, including body weight at endpoint, loading, and treatment. For all muscles, body weight was the most significant predictor of muscle wet mass (*p* < 0.0001), while loading was a significant predictor of soleus wet mass. Based on these findings, hindlimb muscle wet mass has been normalized to endpoint body weight in all animals and subsequently analyzed using a one-way ANOVA followed by Tukey’s post hoc test for multiple comparisons. Results of the ANOVA are displayed in Table 1, while results of post hoc tests are indicated by letters, with groups sharing the same letter considered not significantly different. S: soleus, GP: gastrocnemius and plantaris, TA: tibialis anterior, EDL: extensor digitorum longus.

## Data Availability

The data presented in this study are available upon request.

## Abbreviations

The following abbreviations are used in this manuscript:

ERα	Estrogen receptor alpha
ERβ	Estrogen receptor beta
PWB	Partial weight-bearing
RSV	Resveratrol
HLS	Hindlimb suspension
AUC	Area under curve
AUC LX	Area under curve of longitudinal reactance
½ RT	Half-relaxation time
TTP	Time to peak
TA	Tibialis anterior
EDL	Extensor digitorum longus
BW	Body weight
EIM	Electrical impedance myography
MPP	methyl-piperidino-pyrazole
AKT	Protein kinase B
MuRF 1	Muscle ring finger protein 1
PAX 7	Paired box 7
P-ER	Phosphorylated estrogen receptor
P-AKT	Phosphorylated protein kinase B
FOXO	Forkhead box protein O
DMSO	Dimethyl sulfoxide
RIPA	Radioimmunoprecipitation assay
SEM	Standard error of the mean
GAPDH	Glyceraldehyde-3-phosphate dehydrogenase
AU	Arbitrary units
